# A fast quantum interface between different spin qubit encodings

**DOI:** 10.1038/s41467-018-07522-1

**Published:** 2018-11-29

**Authors:** A. Noiri, T. Nakajima, J. Yoneda, M. R. Delbecq, P. Stano, T. Otsuka, K. Takeda, S. Amaha, G. Allison, K. Kawasaki, Y. Kojima, A. Ludwig, A. D. Wieck, D. Loss, S. Tarucha

**Affiliations:** 1 RIKEN, Center for Emergent Matter Science (CEMS), Wako-shi, Saitama 351-0198 Japan; 2 Laboratoire Pierre Aigrain, Ecole Normale Supérieure-PSL Research University, CNRS, Université Pierre et Marie Curie-Sorbonne Universités, Université Paris Diderot-Sorbonne Paris Cité, 24 rue Lhomond, 75231 Paris Cedex 05, France; 3 Research Institute of Electrical Communication, Tohoku University, 2-1-1 Katahira, Aoba-ku Sendai, 980-8577 Japan; 4 JST, PRESTO, 4-1-8 Honcho, Kawaguchi, Saitama 332-0012 Japan; 5 Department of Applied Physics, University of Tokyo, 7-3-1 Hongo, Bunkyo-ku Tokyo, 113-8656 Japan; 6 Lehrstuhl für Angewandte Festkörperphysik, Ruhr-Universität Bochum, D-44780 Bochum, Germany; 7 Department of Physics, University of Basel, Klingelbergstrasse 82, 4056 Basel, Switzerland

## Abstract

Single-spin qubits in semiconductor quantum dots hold promise for universal quantum computation with demonstrations of a high single-qubit gate fidelity above 99.9% and two-qubit gates in conjunction with a long coherence time. However, initialization and readout of a qubit is orders of magnitude slower than control, which is detrimental for implementing measurement-based protocols such as error-correcting codes. In contrast, a singlet-triplet qubit, encoded in a two-spin subspace, has the virtue of fast readout with high fidelity. Here, we present a hybrid system which benefits from the different advantages of these two distinct spin-qubit implementations. A quantum interface between the two codes is realized by electrically tunable inter-qubit exchange coupling. We demonstrate a controlled-phase gate that acts within 5.5 ns, much faster than the measured dephasing time of 211 ns. The presented hybrid architecture will be useful to settle remaining key problems with building scalable spin-based quantum computers.

## Abstract (web summary)

The race to produce a quantum computer has driven the development of many different qubit designs with different benefits and drawbacks. Noiri et al. demonstrate a hybrid device with two coupled semiconductor spin qubits of different designs, which should allow each qubit’s advantages to be exploited.

## Introduction

Initialization, single-qubit and two-qubit gate operations, and measurements are fundamental elements for universal quantum computation^1^. Generally, they should all be fast and with high fidelity to reach the fault-tolerance thresholds^2^. So far, various encodings of spin qubits into one to three-spin subspaces have been developed in semiconductor quantum dots^3–15^. In particular, recent experiments demonstrated all of these elements including two-qubit logic gates for single-spin qubits proposed by Loss and DiVincenzo (LD qubits) and singlet-triplet (ST) qubits^6–8,14^. These qubits have different advantages depending on the gate operations, and combinations thereof can increase the performance of spin-based quantum computing. In LD qubits, the two-qubit gate is fast^6,7^ as it relies on the exchange interaction between neighboring spins. In contrast, the two-qubit gate in ST qubits is much slower as it is mediated by a weak dipole coupling^14^. Concerning initialization and readout, however, the situation is the opposite: it is slow for LD qubits, relying on spin-selective tunneling to a lead^16,17^, while it is orders of magnitude faster in ST qubits relying on Pauli spin blockade^12,13^. Therefore, a fast and reliable interface between LD and ST qubits would allow for merging the advantages of both realizations.

Here we present such an interface implementing a controlled-phase (CPHASE) gate between a LD qubit and a ST qubit in a quantum dot array^18,19^. The gate is based on the nearest neighbor exchange coupling and is performed in 5.5 ns. Even though we do not pursue benchmarking protocols here, the gate time being much shorter than the corresponding dephasing time (211 ns) indicates that the fidelity of this type of gates can be very high. Our results demonstrate that controlled coherent coupling of different types of gated spin qubits is feasible, and one can proceed to combining their advantages. Overall, our work pushes further the demonstrated scalability of spin qubits in quantum dot arrays.

## Results

### A LD qubit and a ST qubit formed in a triple quantum dot (TQD)

**Fig. 1:**
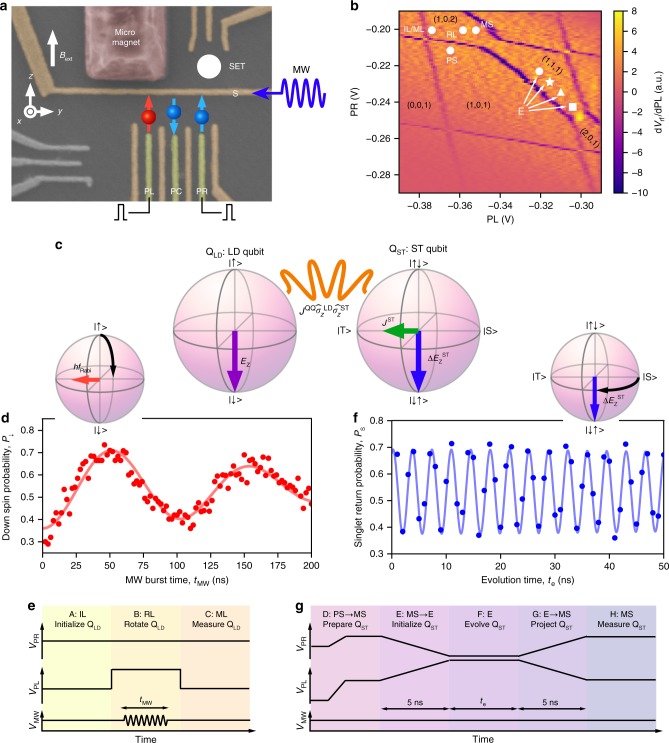
Hybrid system of a LD qubit and a ST qubit realized in a TQD. **a** False color scanning electron microscope image of a device identical to the one used in this study. The TQD is defined in a two-dimensional electron gas at the GaAs/AlGaAs heterointerface 100 nm below the surface. The upper single electron transistor is used for radiofrequency-detected charge sensing^24,25^. A MW with a frequency of 17.26 GHz is applied to the S gate to drive EDSR. **b** Stability diagram of the TQD obtained by differentiating the charge sensing signal *V*_rf_. **c** Hybrid system of a LD qubit and a ST qubit coupled by the exchange coupling *J*^QQ^. **d** Rabi oscillation of Q_LD_ (rotation around *x*-axis) driven by EDSR with *J*^QQ^ ~ 0 at point RL in Fig. 1b. The data is fitted to oscillations with a Gaussian decay of $T_2^{{\mathrm{Rabi}}}$ = 199 ns. **e** Pulse sequence used to produce Fig. 1d showing gate voltages *V*_PL_ and *V*_PR_ applied to the PL and PR gates and a MW burst *V*_MW_. **f** Precession of Q_ST_ (rotation around *z*-axis) with a frequency of *f*^ST^ = 280 MHz due to ${\mathrm{\Delta }}E_{\mathrm{Z}}^{{\mathrm{ST}}}$, taken at point E marked by the white circle in (1,1,1) in Fig. 1b, where *J*^QQ^ and *J*^ST^ ~ 0. The data follow the Gaussian decay with a decay time of 207 ns (see Supplementary Fig. 2a) induced by the nuclear field fluctuations^29^. **g** Pulse sequence used to produce Fig. 1f

A hybrid system comprising a LD qubit and a ST qubit is implemented in a linearly-coupled gate-defined TQD shown in Fig. 1a. The LD qubit (Q_LD_) is formed in the left dot while the ST qubit (Q_ST_) is hosted in the other two dots. We place a micro-magnet near the TQD to coherently and resonantly control Q_LD_ via electric dipole spin resonance (EDSR)^20–23,26^. At the same time it makes the Zeeman energy difference between the center and right dots, ${\mathrm{\Delta }}E_{\mathrm{Z}}^{{\mathrm{ST}}}$, much larger than their exchange coupling *J*^ST^, such that the eigenstates of Q_ST_ become |↑↓〉 and |↓↑〉 rather than singlet |S〉 and triplet |T〉. We apply an external in-plane magnetic field *B*_ext_ = 3.166 T to split the Q_LD_ states by the Zeeman energy *E*_Z_ as well as to separate polarized triplet states |↑↑〉 and |↓↓〉 from the Q_ST_ computational states. The experiment is conducted in a dilution refrigerator with an electron temperature of approximately 120 mK. The qubits are manipulated in the (N_L_, N_C_, N_R_) = (1,1,1) charge state while the (1,0,1) and (1,0,2) charge states are also used for initialization and readout (see Fig. 1b). Here, N_L(C,R)_ denotes the number of electrons inside the left (center, right) dot.

We first independently measure the coherent time evolution of each qubit to calibrate the initialization, control, and readout. We quench the inter-qubit exchange coupling by largely detuning the energies of the (1,1,1) and (2,0,1) charge states. For Q_LD_, we observe Rabi oscillations^4^ with a frequency *f*_Rabi_ of up to 10 MHz (Fig. 1d) as a function of the microwave (MW) burst time *t*_MW_, using the pulse sequence in Fig. 1e. For Q_ST_, we observe the precession between |S〉 and |T〉 (ST precession) (Fig. 1f) as a function of the evolution time *t*_e_, using the pulse sequence in Fig. 1g (see Supplementary Note 2 for full control of Q_ST_). We use a metastable state to measure Q_ST_ with high fidelity^13^ (projecting to |S〉 or |T〉) in the presence of large ${\mathrm{\Delta }}E_{\mathrm{Z}}^{{\mathrm{ST}}}$ with which the lifetime of |T〉 is short^27^.

### Calibration of the two-qubit coupling

**Fig. 2:**
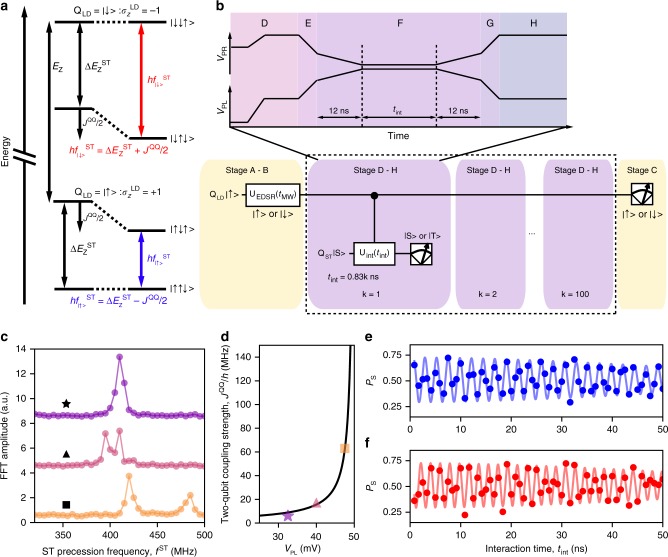
ST qubit frequency controlled by the LD qubit. **a** Energy diagram of the two-qubit states for $E_{\mathrm{Z}} \gg {\mathrm{\Delta }}E_{\mathrm{Z}}^{{\mathrm{ST}}},{\mathrm{\Delta }}E_{\mathrm{Z}}^{{\mathrm{QQ}}} \gg J^{{\mathrm{QQ}}}$ (*J*^ST^ = 0). The ST qubit frequency is equal to ${\mathrm{\Delta }}E_{\mathrm{Z}}^{{\mathrm{ST}}}$ for *J*^QQ^ = 0, and shifts by ±*J*^QQ^/2 depending on the Q_LD_ state for finite *J*^QQ^. **b** The quantum circuit for demonstrating the phase control of Q_ST_ depending on Q_LD_. After preparing an arbitrary state of Q_LD_ (stages A and B), we run modified stages from D to H (shown in the upper panel) 100 times with *t*_int_ values ranging from 0.83 to 83 ns to observe the time evolution of Q_ST_ without reinitializing or measuring Q_LD_. Stages A, B and C take 202 μs in total and the part from D to H is 7 μs long. **c** FFT spectra of *f*^ST^ with different interaction points shown by the white corresponding symbol in Fig. 1b (traces offset for clarity). In addition to the frequency splitting due to *J*^QQ^, the center frequency of the two peaks shifts because ${\mathrm{\Delta }}E_{\mathrm{Z}}^{{\mathrm{ST}}}$ is also dependent on the interaction point (Methods). **d** Interaction point dependence of the ST qubit frequency splitting, i.e. the two-qubit coupling strength *J*^QQ^/*h*, fitted with the black model curve (see Supplementary Note 4 for the data extraction and fitting). **e** ST precession for the Q_LD_ input state |↑〉 fitted with the Gaussian-decaying oscillations with a decay time of 72 ns. **f** ST precession for the Q_LD_ input state |↓〉 with a fitting curve. The decay time is 75 ns. The total data acquisition time for **e** and **f** is 451 ms

The two qubits are interfaced by exchange coupling *J*^QQ^ between the left and center dots as illustrated in Fig. 1c. We operate the two-qubit system under the conditions of $E_{\mathrm{Z}} \gg {\mathrm{\Delta }}E_{\mathrm{Z}}^{{\mathrm{ST}}},{\mathrm{\Delta }}E_{\mathrm{Z}}^{{\mathrm{QQ}}} \gg J^{{\mathrm{QQ}}} \gg J^{{\mathrm{ST}}}$ where ${\mathrm{\Delta }}E_{\mathrm{Z}}^{{\mathrm{QQ}}}$ is the Zeeman energy difference between the left and center dots. Then, the Hamiltonian of the system is

1 $${\cal H} = - E_{\mathrm{Z}}\hat \sigma _z^{{\mathrm{LD}}}/2 - {\mathrm{\Delta }}E_{\mathrm{Z}}^{{\mathrm{ST}}}\hat \sigma _z^{{\mathrm{ST}}}/2 + J^{{\mathrm{QQ}}}(\hat \sigma _z^{{\mathrm{LD}}}\hat \sigma _z^{{\mathrm{ST}}} - 1)/4$$

where $\hat \sigma _z^{{\mathrm{LD}}}$ and $\hat \sigma _z^{{\mathrm{ST}}}$ are the Pauli z-operators of Q_LD_ and Q_ST_, respectively^18^ (Supplementary Note 3). The last term in Eq. (1) reflects the effect of the inter-qubit coupling *J*^QQ^: for states in which the spins in the left and center dots are antiparallel, the energy decreases by *J*^QQ^/2 (see Fig. 2a). In the present work, we choose to operate Q_LD_ as a control qubit and Q_ST_ as a target, although these are exchangeable. With this interpretation, the ST precession frequency *f*^ST^ depends on the state of Q_LD,_ $f_{\sigma _z^{{\mathrm{LD}}}}^{{\mathrm{ST}}} = \left( {{\mathrm{\Delta }}E_{\mathrm{Z}}^{{\mathrm{ST}}} - \sigma _z^{{\mathrm{LD}}}J^{{\mathrm{QQ}}}/2} \right)/h$. Here $\sigma _z^{{\mathrm{LD}}}$ represents |↑〉 or |↓〉 and +1 or −1 interchangeably. This means that while *J*^QQ^ is turned on for the interaction time *t*_int_, Q_ST_ accumulates the controlled-phase *ϕ*_C_ = 2*πJ*^QQ^*t*_int_/*h*, which provides the CPHASE gate (up to single-qubit phase gates; see Supplementary Note 7) in *t*_int_ = *h*/2*J*^QQ^. An important feature of this two-qubit gate is that it is intrinsically fast, scaling with *J*^QQ^/*h* which can be tuned up to ~100 MHz, and is limited only by the requirement $J^{{\mathrm{QQ}}}/h \ll {\mathrm{\Delta }}E_{\mathrm{Z}}^{{\mathrm{QQ}}}/h \sim 500\,{\mathrm{MHz}}$ in our device.

Before testing the two-qubit gate operations, we calibrate the inter-qubit coupling strength *J*^QQ^, and its tunability by gate voltages. The inter-qubit coupling in pulse stage F (Fig. 2b) is controlled by the detuning energy between (2,0,1) and (1,1,1) charge states (one of the points denoted E in Fig. 1b). To prevent leakage from the Q_ST_ computational states, we switch *J*^QQ^ on and off adiabatically with respect to ${\mathrm{\Delta }}E_{\mathrm{Z}}^{{\mathrm{QQ}}}$ by inserting voltage ramps to stage F with a total ramp time of *t*_ramp_ = 24 ns (Fig. 2b)^28^. The coherent precession of Q_ST_ is measured by repeating the pulse stages from D to H without initializing, controlling and measuring Q_LD_, which makes Q_LD_ a random mixture of |↑〉 and |↓〉. Figure 2c shows the FFT spectra of the precession measured for various interaction points indicated in Fig. 1b. As we bring the interaction point closer to the boundary of (1,1,1) and (2,0,1), *J*^QQ^ becomes larger and we start to see splitting of the spectral peaks into two. The separation of the two peaks is given by *J*^QQ^/*h* which can be controlled by the gate voltage as shown in Fig. 2d.

We now demonstrate the controllability of the ST precession frequency by the input state of Q_LD_, the essence of a CPHASE gate. We use the quantum circuit shown in Fig. 2b, which combines the pulse sequences for independent characterization of Q_LD_ and Q_ST_. Here we choose the interaction point such that *J*^QQ^/*h* = 90 MHz. By using either |↑〉 or |↓〉 as the Q_LD_ initial state (the latter prepared by an EDSR *π* pulse), we observe the ST precessions as shown in Fig. 2e, f. The data fit well to Gaussian-decaying oscillations giving $f_{| \uparrow \rangle}^{{\mathrm{ST}}} = 434 \pm 0.5\,{\mathrm{MHz}}$ and $f_{| \downarrow \rangle}^{{\mathrm{ST}}} = 524 \pm 0.4\,{\mathrm{MHz}}$ [These are consistent with the values determined by Bayesian estimation discussed in Methods]. This demonstrates the control of the precession rate of Q_ST_ by *J*^QQ^/*h* depending on the state of Q_LD_.

### Demonstration of a CPHASE gate

**Fig. 3:**
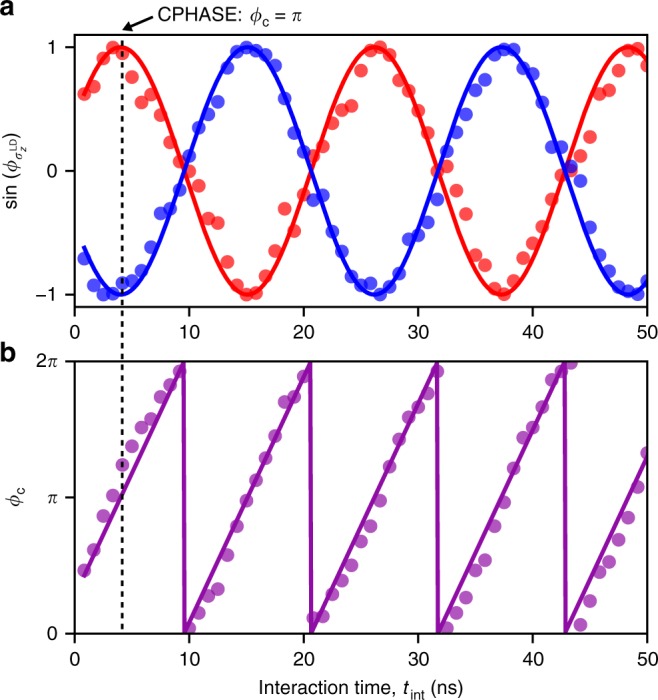
Controlled-phase evolution. **a** Interaction time *t*_int_ dependence of $\phi _{\sigma _z^{{\mathrm{LD}}}}$ controlled by Q_LD_. The blue and red data are for Q_LD_ = |↑〉 and |↓〉, respectively. The solid curves are sin(*πJ*^QQ^(*t*_int_ + *t*_0_)/*h*) (red) and sin(−*πJ*^QQ^(*t*_int_ + *t*_0_)/*h*) (blue) where the values of *J*^QQ^ and *t*_0_ are obtained in the MLE. The curves are consistent with the data as expected. **b** Controlled-phase *ϕ*_C_ = *ϕ*_|↓〉_ − *ϕ*_|↑〉_ extracted from Fig. 3a. Including the initial phase accumulated during gate voltage ramps at stage F, *ϕ*_C_ reaches *π* first at *t*_int_ = 4.0 ns and increases by *π* in every 5.5 ns afterwards

To characterize the controlled-phase accumulated during the pulse stage F, we separate the phase of Q_ST_ into controlled and single-qubit contributions as $\phi _{\sigma _z^{{\mathrm{LD}}}} = - \pi \sigma _z^{{\mathrm{LD}}}J^{{\mathrm{QQ}}}\left( {t_{{\mathrm{int}}} + t_0} \right)/h$ and $\phi ^{{\mathrm{ST}}} = 2\pi {\mathrm{\Delta }}E_{\mathrm{Z}}^{{\mathrm{ST}}}( {t_{{\mathrm{int}}} + t_{{\mathrm{ramp}}}} )/h + \phi _0$, respectively. Here *t*_0 _(≪*t*_ramp_) represents the effective time for switching on and off *J*^QQ^ (Supplementary Note 5). A phase offset *ϕ*_0_ denotes the correction accounting for nonuniform ${\mathrm{\Delta }}E_{\mathrm{Z}}^{{\mathrm{ST}}}$ during the ramp (Supplementary Note 5). Then the probability of finding the final state of Q_ST_ in singlet is modeled as

2 $$P_{{\mathrm{S}},{\mathrm{model}}} = a{\mathrm{cos}}\left( {\phi _{\sigma _z^{{\mathrm{LD}}}} + \phi ^{{\mathrm{ST}}}} \right)\exp \left( { - (t_{{\mathrm{int}}}/T_2^ \ast )} \right)^2 + b$$

where *a*, *b* and $T_2^ \ast$ represent the values of amplitude, mean and the dephasing time of the ST precession, respectively. We use maximum likelihood estimation (MLE) combined with Bayesian estimation^29,30^ to fit all variables in Eq. 2, that are $a,b,t_0,J^{{\mathrm{QQ}}},T_2^ \ast ,\phi _0$, and ${\mathrm{\Delta }}E_{\mathrm{Z}}^{{\mathrm{ST}}}$, from the data (Methods). This allows us to extract the *t*_int_ dependence of $\phi _{\sigma _z^{{\mathrm{LD}}}}$ (Fig. 3a) (Methods) and consequently *ϕ*_C_ = *ϕ*_*|*↓〉_ − *ϕ*_|↑〉_ (Fig. 3b). It evolves with *t*_int_ in the frequency of *J*^QQ^/*h* = 90 MHz, indicating that the CPHASE gate time can be as short as *h*/2*J*^QQ^ = 5.5 ns (up to single-qubit phase). On the other hand, $T_2^ \ast$ obtained in the MLE is 211 ns, much longer than what is observed in Fig. 2e, f because the shorter data acquisition time used here cuts off the low-frequency component of the noise spectrum^29^. We note that this $T_2^ \ast$ is that for the two-qubit gate while *J*^QQ^ is turned on^8^, and therefore it is likely to be dominated by charge noise rather than the nuclear field fluctuation (Supplementary Note 6). The ratio $2J^{{\mathrm{QQ}}}T_2^ \ast /h$ suggests that 38 CPHASE operations would be possible within the two-qubit dephasing time. We anticipate that this ratio can be further enhanced by adopting approaches used to reduce the sensitivity to charge noise in exchange gates such as symmetric operation^31,32^ and operation in an enhanced field gradient^33^.

**Fig. 4:**
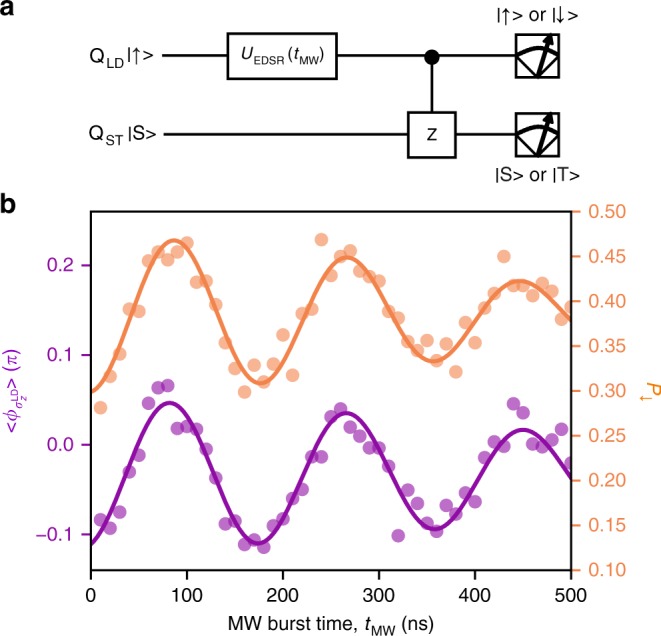
Demonstration of the controlled-phase gate for arbitrary control qubit states. **a** The circuit for CPHASE gate demonstration. Here *t*_int_ is fixed at 4.2 ns where *ϕ*_C_ ≈ *π* (Fig. 3b). **b** *t*_MW_ dependence of the spin-down probability of Q_LD_, *P*_↓_ (yellow) and the averaged $\phi _{\sigma _z^{{\mathrm{LD}}}}$, $\left\langle \phi _{\sigma _z^{{\mathrm{LD}}}} \right\rangle$ (purple) obtained by the circuit shown in Fig. 4a. $\left\langle \phi _{\sigma _z^{{\mathrm{LD}}}} \right\rangle$ $\left( { = - \pi \langle \sigma _z^{{\mathrm{LD}}} \rangle /2} \right)$ is expected to be proportional to *P*_↓_. We see $\left\langle \phi _{\sigma _z^{{\mathrm{LD}}}} \right\rangle$ oscillates depending on the input Q_LD_ state. The oscillation visibility of $\left\langle \phi _{\sigma _z^{{\mathrm{LD}}}} \right\rangle$ is most probably limited by low preparation fidelity of the input Q_LD_ state as the visibility of the oscillation in *P*_↓_ is also low (see Methods)

Finally we show that the CPHASE gate operates correctly for arbitrary Q_LD_ input states. We implement the circuit shown in Fig. 4a in which *t*_int_ is fixed to yield *ϕ*_C_ = *π*, while a coherent initial Q_LD_ state with an arbitrary $\sigma _z^{{\mathrm{LD}}}$ is prepared by EDSR. We extract the averaged $\phi _{\sigma _z^{{\mathrm{LD}}}}$, $\left\langle \phi _{\sigma _z^{{\mathrm{LD}}}} \right\rangle$ by Bayesian estimation^29,30^, which shows an oscillation as a function of *t*_MW_ in agreement with the Rabi oscillation measured independently by reading out Q_LD_ at stage C as shown in Fig. 4b (see Methods for the estimation procedure and the origin of the low visibility, i.e., ${\mathrm{max}}| {\langle {\phi _{\sigma _z^{{\mathrm{LD}}}}} \rangle } | < \pi /2$). These results clearly demonstrate the CPHASE gate functioning for an arbitrary Q_LD_ input state.

## Discussion

In summary, we have realized a fast quantum interface between a LD qubit and a ST qubit using a TQD. The CPHASE gate between these qubits is performed in 5.5 ns, much faster than its dephasing time of 211 ns and those ratio (~38) would be high enough to provide a high-fidelity CPHASE gate (Supplementary Note 8). Optimizing the magnet design to enhance the field gradient would allow even faster gate time beyond GHz with larger *J*^QQ^. At the same time, this technique is directly applicable to Si-based devices with much better single-qubit coherence^5–9^. Our results suggest that the performance of certain quantum computational tasks can be enhanced by adopting different kinds of qubits for different roles. For instance, LD qubits can be used for high-fidelity control and long memory and the ST qubit for fast initialization and readout. This combination is ideal for example, the surface code quantum error correction where a data qubit must maintain the coherence while a syndrome qubit must be measured quickly^34^. Furthermore, the fast (~100 ns^25^) ST qubit readout will allow the read out of a LD qubit in a quantum-non-demolition manner^35^ with a speed three orders of magnitude faster than a typical energy-selective tunneling measurement^16,17^. Viewed from the opposite side, we envisage coupling two ST qubits through an intermediate LD qubit, which would boost the two ST qubit gate speed by orders of magnitude compared to the demonstrated capacitive coupling scheme^14^. In addition, our results experimentally support the concept of the theoretical proposal of a fast two-qubit gate between two ST qubits based on direct exchange^36^ which shares the same working principle as our two-qubit gate. Our approach will further push the demonstrated scalability of spin qubits in quantum dot arrays beyond the conventional framework based on a unique spin-qubit encoding.

## Methods

### Device design

Our device was fabricated on a GaAs/Al_0.3_Ga_0.7_As heterostructure wafer having a two-dimensional electron gas 100 nm below the surface, grown by molecular beam epitaxy on a semi-insulating (100) GaAs substrate. The electron density *n* and mobility *μ* at a temperature of 4.2 K are *n* = 3.21 × 10^15^ m^−2^ and *μ* = 86.5 m^2^ V^−1^ s^−1^ in the dark, respectively. We deposited Ti/Au gate electrodes to define the TQD and the charge sensing single electron transistor. A piece of Co metal (micro-magnet, MM) is directly placed on the surface of the wafer to provide a local magnetic field gradient in addition to the external magnetic field applied in-plane (along *z*). The MM geometry is designed based on the numerical simulations of the local magnetic field^23^. The field property is essentially characterized by the two parameters^23^: d*B*_*x*_/d*z* at the position of each dot and the difference in *B*_*z*_ between the neighboring dots, Δ*B*_*z*_ (see Fig. 1a for the definition of the *x* and *z* axes). d*B*_*x*_/d*z* determines the spin rotation speed by EDSR and is as large as ~1 mT nm^−1^ at the left dot (Supplementary Fig. 5a) allowing fast control of Q_LD_ (*f*_Rabi_ > 10 MHz)^20,23^. At the same time Δ*B*_*z*_ between the left and center dots, ${\mathrm{\Delta }}B_z^{{\mathrm{LC}}}$, is designed to be ~60 mT (Supplementary Fig. 5b) to guarantee the selective EDSR control of Q_LD_ without rotating the spin in the center dot^20,23^. Furthermore, Δ*B*_*z*_ between the center and right dots, ${\mathrm{\Delta }}B_z^{{\mathrm{CR}}}$, is designed to be ~40 mT (Supplementary Fig. 5b) to make the eigenstates of Q_ST_ |↑↓〉 and |↓↑〉 rather than |S〉 and |T〉 by satisfying ${\mathrm{\Delta }}E_{\mathrm{Z}}^{{\mathrm{ST}}} \gg J^{{\mathrm{ST}}}$. Note that ${\mathrm{\Delta }}E_{\mathrm{Z}}^{{\mathrm{ST}}} = |g|\mu _{\mathrm{B}}{\mathrm{\Delta }}B_z^{{\mathrm{CR}}}$ where *g* *~* −0.4 and *μ*_B_ are the electron *g*-factor and Bohr magneton, respectively. From the design we expect a large variation of ${\mathrm{\Delta }}B_z^{{\mathrm{CR}}}$ when the electron in the center dot is displaced by the electric field. Indeed, we observe a strong influence of the gate voltages on ${\mathrm{\Delta }}B_z^{{\mathrm{CR}}}$, which reaches ~100 mT $\left( {{\mathrm{\Delta }}E_{\mathrm{Z}}^{{\mathrm{ST}}}/h \sim 500\,{\mathrm{MHz}}} \right)$ in the configuration chosen for the two-qubit gate experiment.

### Estimation of the ST precession parameters

Here we describe the estimation of the ST precession parameters in Eq. 2 under the influence of a fluctuating single-qubit phase of Q_ST_. Out of the parameters involved, $\phi _{\sigma _z^{{\mathrm{LD}}}}$ is the only parameter assumed to be Q_LD_ state-dependent, and the rest is classified into two types. One is the pulse-cycle-independent parameters, $a,b,J^{{\mathrm{QQ}}},T_2^ \ast$ and *t*_0_ which is constant during the experiment, and the other is the pulse-cycle-dependent parameters, $\sigma _z^{{\mathrm{LD}}},{\mathrm{\Delta }}E_{\mathrm{Z}}^{{\mathrm{ST}}}$ and *ϕ*_0_, which can change cycle by cycle. Each pulse cycle consists of pulse stages from A to C as shown in Fig. 2b. We run the pulse cycle consecutively with a MW frequency fixed at 17.26 GHz and collect the data while Q_LD_ drifts between on-resonances and off-resonances with the MW burst due to the nuclear field fluctuation. To decrease the uncertainty of the estimated parameters, we choose the cycles during which the spin flip of Q_LD_ is unlikely in the following manner. The cycles throughout which Q_LD_ is likely to be |↓〉 are post-selected by the condition that Q_LD_ is on-resonance (i.e., Rabi oscillation of Q_LD_ is observed in ensemble-averaged data from nearby cycles) and the final state of Q_LD_ is measured to be |↓〉 at pulse stage C. Similarly, the cycles for Q_LD_ = |↑〉 are post-selected by the condition that Q_LD_ is off-resonance and the final state of Q_LD_ is measured to be |↑〉. The data structure and the index definitions for MLE are summarized in Supplementary Table 1. *k* is the index of the interaction time such that *t*_int_ = 0.83 × *k* ns with *k* ranging from 1 to 100. *m* is the pulse-cycle index ranging from 1 (2001) to 2000 (4000) for Q_LD_ prepared in |↑〉 (|↓〉). The estimation procedure is the following. From all the readout results of Q_ST_ (stage H) obtained in the cycles, we first estimate the five pulse-cycle-independent parameters by MLE. Note that *J*^QQ^ may have a small pulse-cycle-dependent component due to charge noise but this effect is captured as additional fluctuation in ${\mathrm{\Delta }}E_{\mathrm{Z}}^{{\mathrm{ST}}}$ and *ϕ*_0_ in our model. We apply MLE to 100 × 4000 readout results of Q_ST_, $r_m^k = 1$ (0) for Q_ST_ = |S〉 (|T〉). To this end, we first introduce the likelihood *P*_*m*_ defined in the eight dimensional parameter space as

3 $$P_m\left( {a,b,t_0,J^{{\mathrm{QQ}}},T_2^ \ast ,\sigma _z^{{\mathrm{LD}}},\phi _0,{\mathrm{\Delta }}E_{\mathrm{Z}}^{{\mathrm{ST}}}} \right) = \mathop {\prod }\limits_{k = 1}^{100} \left( {r_m^kP_{{\mathrm{S}},{\mathrm{model}}} + (1 - r_m^k)(1 - P_{{\mathrm{S}},{\mathrm{model}}})} \right)$$

where *P*_S,model_ is defined in Eq. (2). We calculate *P*_*m*_ on a discretized space within a chosen parameter range (Supplementary Table 2) using a single cycle data. Then we obtain *P*_*m*_ for the target five parameters as a marginal distribution by tracing out the pulse-cycle-dependent parameters,

4 $$P_{\mathrm{m}}\left( {a,b,t_0,J^{{\mathrm{QQ}}},T_2^ \ast } \right) = \mathop {\sum }\limits_{\sigma _z^{{\mathrm{LD}}}} \mathop {\sum }\limits_{\phi _0} \mathop {\sum }\limits_{{\mathrm{\Delta }}E_{\mathrm{Z}}^{{\mathrm{ST}}}} P_m\left( {a,b,t_0,J^{{\mathrm{QQ}}},T_2^ \ast ,\sigma _z^{{\mathrm{LD}}},\phi _0,{\mathrm{\Delta }}E_{\mathrm{Z}}^{{\mathrm{ST}}}} \right).$$

Repeating this process for all pulse cycles, we obtain the likelihood *P* as

5 $$P\left( {a,b,t_0,J^{{\mathrm{QQ}}},T_2^ \ast } \right) = \mathop {\prod }\limits_m P_m\left( {a,b,t_0,J^{{\mathrm{QQ}}},T_2^ \ast } \right).$$

We choose the maximum of *P* as the estimator for *a*, *b*, *t*_0_,* J*^QQ^ and $T_2^ \ast$, obtaining *a* = 0.218 ± 0.005, *b* = 0.511 ± 0.003, *t*_0_ = 1.53 ± 0.17 ns, *J*^QQ^/*h* = 90.2 ± 0.3 MHz, $T_2^ \ast = 211 \pm 37$ ns.

Once these values are fixed, we estimate the pulse-cycle-dependent parameters, $\sigma _z^{{\mathrm{LD}}},\phi _0$ and ${\mathrm{\Delta }}E_{\mathrm{Z}}^{{\mathrm{ST}}}$, for each cycle *m*. Note that $\sigma _z^{{\mathrm{LD}}}$ could be prepared deterministically if the state preparation of Q_LD_ were ideal, but here we treat it as one of the parameters to be estimated because of a finite error in the Q_LD_ state preparation. We again evaluate the likelihood $P_m\left( {\sigma _z^{{\mathrm{LD}}},\phi _0,{\mathrm{\Delta }}E_{\mathrm{Z}}^{{\mathrm{ST}}}} \right)$ defined in a discretized three dimensional space of its parameters using Eq. 3 and find their values that maximize the likelihood.

Based on the values of $a,b,T_2^ \ast$ and *ϕ*^ST^ determined above, we can directly estimate $\phi _{\sigma _z^{{\mathrm{LD}}}}$ controlled by Q_LD_ for each *t*_int_ without presumptions on the value of *J*^QQ^. To this end, we search for the parameter $\phi _{\sigma _z^{{\mathrm{LD}}}}$ that maximizes the likelihood

6 $$P^k\left( {\phi _{\sigma _z^{{\mathrm{LD}}}}} \right) = \mathop {\prod }\limits_m \left( {r_m^kP_{{\mathrm{S}},{\mathrm{model}}} + \left( {1 - r_m^k} \right)\left( {1 - P_{{\mathrm{S}},{\mathrm{model}}}} \right)} \right).$$

The obtained estimators for *ϕ*_|↓〉_ and *ϕ*_|↑〉_ are consistent with the expected values ±*πJ*^QQ^(*t*_int_ + *t*_0_)/*h* calculated from *J*^QQ^/*h* and *t*_0_ found above (see Fig. 3a).

The ensemble-averaged phase $\left\langle {\phi _{\sigma _z^{{\mathrm{LD}}}}} \right\rangle$ is obtained based on a similar estimation protocol. Here we estimate $\phi _{\sigma _z^{{\mathrm{LD}}}}$ for each *m* with fixed *k* = 5 (*t*_int_ = 4.2 ns) to yield *ϕ*_C_ ≈ *π* from the likelihood $P_m^{k = 5} = r_m^{k = 5}P_{{\mathrm{S}},{\mathrm{model}}} + \left( {1 - r_m^{k = 5}} \right)\left( {1 - P_{{\mathrm{S}},{\mathrm{model}}}} \right)$ and then take the average of the estimated values for 800 pulse cycles. The oscillation visibility of $\left\langle {\phi _{\sigma _z^{{\mathrm{LD}}}}} \right\rangle$ in Fig. 4b is limited by three factors, low preparation fidelity of the input Q_LD_ state, estimation error of $\phi _{\sigma _z^{{\mathrm{LD}}}}$ and CPHASE gate error. The first contribution is likely to be dominant as the visibility of the oscillation in P_↓_ is correspondingly low. Note that the effect of those errors is not visible in Fig. 3 because the most likely values of $\phi _{\sigma _z^{{\mathrm{LD}}}}$ are plotted.

## Electronic supplementary material

Supplementary Information file

Peer Review file

## Data Availability

The data that support the findings of this study are available from the corresponding authors upon reasonable request.
