# Supplementary material for: A fast quantum interface between different spin qubit encodings
Source: Nat Commun. 2018 Nov 29;9:5066. doi: 10.1038/s41467-018-07522-1 (PMC6265340; doi:10.1038/s41467-018-07522-1)
Supplement: Supplementary file 1 — Supplementary Information file [file 41467_2018_7522_MOESM1_ESM.pdf]

# A fast quantum interface between different spin qubit encodings

Noiri *et al*.

**Supplementary Note 1: The Zeeman energy difference between the nighboring dots.**

To confirm that the oscillation observed in Fig. 1f is driven by $\Delta E_{\mathrm{Z}}^{\mathrm{ST}}$, we measure the EDSR spectrum of the center and right dots. We prepare the doubly-occupied singlet state in the right dot and transfer one of the electrons to the center dot adiabatically with respect to $\Delta E_{\mathrm{Z}}^{\mathrm{ST}}$ to initialize the two spins to $|\uparrow\downarrow\rangle$[1]. Then we measure an EDSR spectrum by frequency chirping with a depth of 10 MHz and MW burst length of 20 μs[2]. Here the spectrum is taken at the same gate voltage configuration as the one used for taking data in Fig. 1f. Then the two-spin state is measured by Pauli spin blockade[1]. When one of the spins rotates and the two-spin state becomes either one of the polarized triplets, the two electrons remain blocked from returning to the doubly-occupied singlet. Supplementary Fig. 1a shows the MW frequency $f_{\mathrm{MW}}$ dependence of the singlet-return probability $\mathrm{P_S}$ with $B_{\mathrm{ext}} = 3.105$ T. The two dips around $f_{\mathrm{MW}} \sim 16.1$ GHz and 16.4 GHz correspond to the resonances of the right and center dots, respectively, which are separated by $\sim$300 MHz in agreement with the ST precession frequency $f^{\mathrm{ST}}$ in Fig. 1f. Because of the strong gate-voltage dependence of $\Delta E_{\mathrm{Z}}^{\mathrm{ST}}$ (see Methods Sec. 1) the obtained $f^{\mathrm{ST}}$ in this measurement is much smaller than that measured in the two-qubit gate experiment ($\Delta E_{\mathrm{Z}}^{\mathrm{ST}}/h = (f_{|\uparrow\rangle}^{\mathrm{ST}} + f_{|\downarrow\rangle}^{\mathrm{ST}})/2 \sim 480$ MHz, see Figs. 2e and 2f).

We also measure the EDSR spectrum of the left and center dots with $B_{\mathrm{ext}} = 3.15$ T as shown in Supplementary Fig. 1b. The two dips of $\mathrm{P_S}$ around $f_{\mathrm{MW}} \sim 17.1$ GHz and 17.6 GHz correspond to the resonances of the center and left dots, respectively. As a consequence, we obtain $\Delta E_{\mathrm{Z}}^{\mathrm{QQ}}/h \sim$500 MHz.

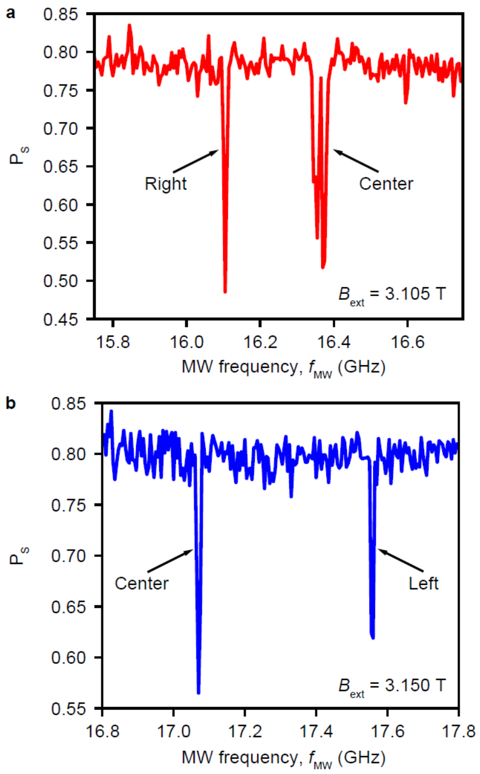


**Supplementary Figure 1 | EDSR spectrum measured between the neighboring dots.**
**a**, EDSR spectrum for the center and right dots taken at the same configuration as the one to obtain the data shown in Fig. 1f. The resonance frequency of the center dot is higher than that of the right dot as the local Zeeman field of the center dot induced by the micro-magnet is larger than that of the right dot (Supplementary Fig. 5b). The separation of the resonance condition ($\sim 300$ MHz) is consistent with the ST precession frequency $f^{\mathrm{ST}}$ in Fig. 1f. **b**, EDSR spectrum for the left and center dots. The resonance frequencies are separated by $\sim 500$ MHz.

## Supplementary Note 2: Full control of $Q_{\mathrm{ST}}$

A set of universal quantum gates in a two-qubit system can be constructed by a CPHASE gate and arbitrary single-qubit gates for each qubit. Arbitrary single-qubit gate operations for $Q_{\mathrm{LD}}$ can be realized by EDSR (see Fig. 1d). In this section we demonstrate full control of $Q_{\mathrm{ST}}$ for completeness. In the following experiment, we quench $J^{\mathrm{QQ}}$ to decouple $Q_{\mathrm{ST}}$ from $Q_{\mathrm{LD}}$.

The rotation of $Q_{\mathrm{ST}}$ around $z$-axis and $x$-axis is mediated by $\Delta E_Z^{\mathrm{ST}}$ and $J^{\mathrm{ST}}$, respectively, as shown in Fig. 1c. Supplementary Fig. 2a shows the precession around $z$-axis which is measured by initializing $Q_{\mathrm{ST}}$ to $|S\rangle$ and projecting the final state along $x$-axis ($|S\rangle$ or $|T\rangle$) using a pulse sequence shown in Fig. 1g. During the evolution in stage F, $J^{\mathrm{ST}}$ is quenched ($\Delta E_Z^{\mathrm{ST}} \gg J^{\mathrm{ST}}$) as the evolution point is far detuned from the resonance of (1,1,1) and (1,0,2) charge states. The fit with the Gaussian decaying

oscillation curve gives $f^{\mathrm{ST}} = 280$ MHz, which is consistent with the FFT spectrum in Supplementary Fig. 2b. Here, the precession visibility is mainly influenced by the error during the state preparation and measurement. The dominant error source is likely the incomplete nonadiabaticity during the state transfers in pulse stages E (for state preparation) and G (for measurement) [the error in charge state discrimination is negligible as described in the main text]. To keep the adiabaticity with respect to the inter-dot tunnel coupling, we choose the pulse rise/fall time of 5 ns, which is too slow to switch $J^{\mathrm{ST}}$ nonadiabatically against $\Delta E_{\mathrm{Z}}^{\mathrm{ST}}/h \sim 280$ MHz. As a result, the prepared state in stage E is inclined to $|\uparrow\downarrow\rangle$ from $|S\rangle$, decreasing the ST precession visibility even though the system is coherent. This error could be suppressed by, instead, initializing to $|\uparrow\downarrow\rangle$ and subsequently rotating around $y$-axis[3,4], although we do not experimentally pursue this alternative preparation. We also note that the ST precession visibility can decrease due to the state leakage to non-qubit states during turning on and off $J^{\mathrm{QQ}}$. We avoid this problem by adiabatically turning on and off $J^{\mathrm{QQ}}$ with respect to $\Delta E_{\mathrm{Z}}^{\mathrm{QQ}}$ (see Supplementary Note 3) and obtain a similar visibility of the ST precessions measured in $J^{\mathrm{QQ}}$ on (Figs. 2e and 2f) and off (Fig. 1f).

Next we demonstrate the qubit control around $x$-axis using a pulse sequence shown in Supplementary Fig. 2d. Here $Q_{\mathrm{ST}}$ is initialized to $|\uparrow\downarrow\rangle$ by slow adiabatic passage, kept at a detuned point (stage F) and projected along $z$-axis ($(|\uparrow\downarrow\rangle$ or $|\downarrow\uparrow\rangle)$) using the reverse process of the initialization[1,5]. Supplementary Fig. 2e shows the evolution of $Q_{\mathrm{ST}}$ as a function of the detuned point. As the point approaches the resonance of (1,1,1) and (1,0,2), $J^{\mathrm{ST}}$ increases and the rotation axis is inclined toward the $x$ direction, eventually realizing the rotation around the $x$-axis for $\Delta E_{\mathrm{Z}}^{\mathrm{ST}} \ll J^{\mathrm{ST}}$. In this scheme, however, the dephasing time decreases as $J^{\mathrm{ST}}$ is increased due to the enhanced exchange noise[6]. To improve the control quality, we employ resonantly driven rotation of $Q_{\mathrm{ST}}$ around the $x$-axis in the rotating frame[3,4]. Here we choose a detuned point where $\Delta E_{\mathrm{Z}}^{\mathrm{ST}} > J^{\mathrm{ST}}$ is satisfied and modulate $J^{\mathrm{ST}}$ at the qubit resonance frequency, $\sqrt{{J^{\mathrm{ST}}}^2 + {\Delta E_{\mathrm{Z}}^{\mathrm{ST}}}^2}/h$, by applying a MW burst to PC gate (see Fig. 1a). Then $Q_{\mathrm{ST}}$ exhibits the Rabi rotation at a frequency proportional to the modulation amplitude. Supplementary Fig. 2g shows the MW frequency dependence of the Rabi oscillations. We find the resonance at $f_{\mathrm{MW}}^{\mathrm{ST}} = 345$ MHz, where we see a clear coherent oscillation as shown in Supplementary Fig. 2h. These results demonstrate full control of $Q_{\mathrm{ST}}$ and therefore, our system is capable of universal two-qubit manipulations.

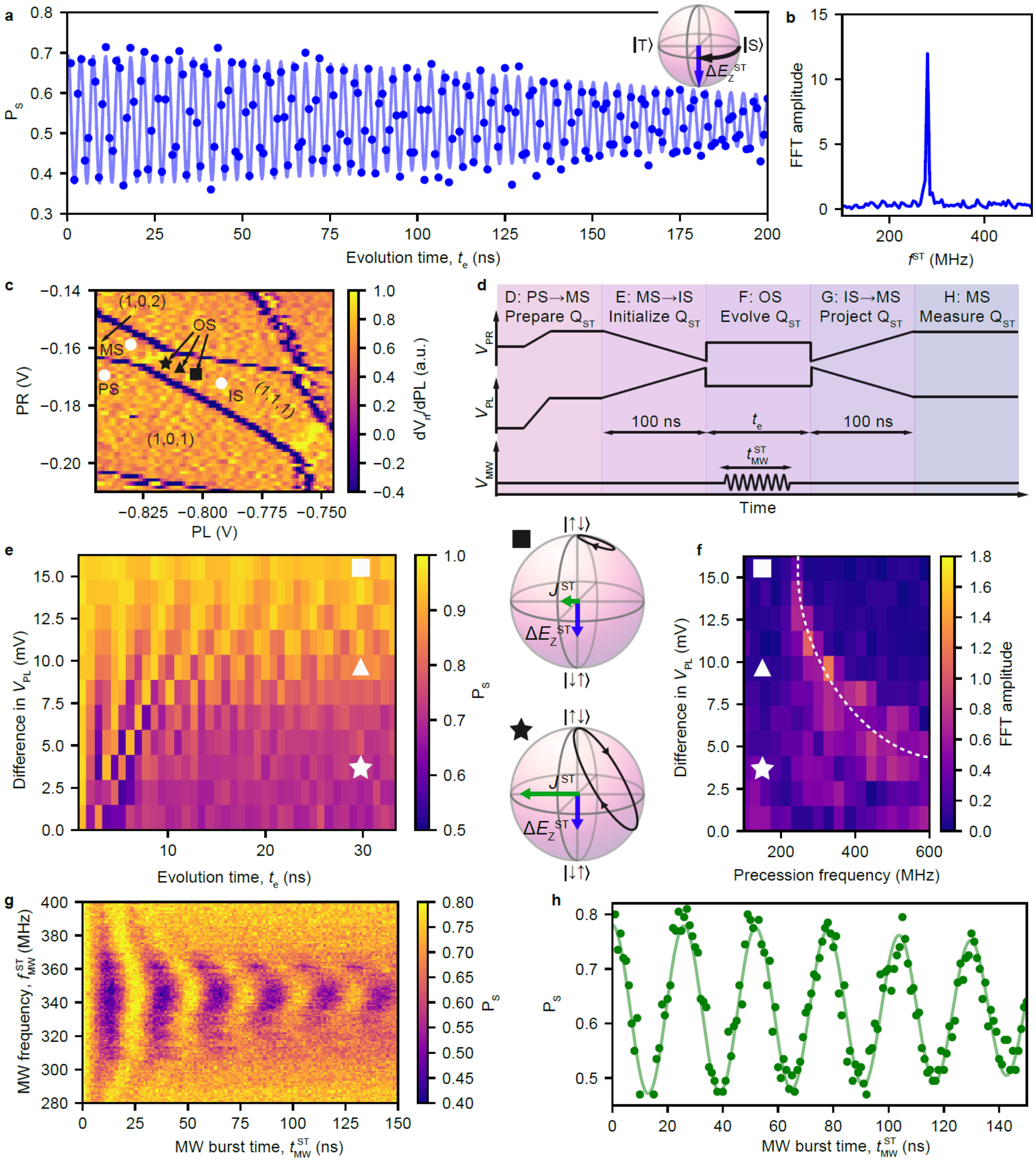


**Supplementary Figure 2 | Full control of $Q_{ST}$.**

**a**, Precession around the *z*-axis due to $\Delta E_Z^{ST}$ by quenching $J^{ST}$. The data and the fitting curve is the same as the one shown in Fig. 1f. The fitting gives the dephasing time of $207 \pm 11$ ns. **b**, FFT spectrum of **a** showing a peak at $f^{ST} = 280$ MHz in agreement with the fitting (see Fig. 1f). **c**, Stability diagram of the TQD used for demonstrating the full control of $Q_{ST}$. [The gate voltage configuration differs from Fig. 1b and the data were taken in a different cool-downs]. **d**, Pulse sequence used to demonstrate the control of $Q_{ST}$ around *x*-axis by $J^{ST}$. In stages E and G, $|\uparrow\downarrow\rangle$ and $|S\rangle$ ($|\downarrow\uparrow\rangle$ and $|T\rangle$) are interconverted adiabatically, allowing a *z*-axis readout of $Q_{ST}$ by the Pauli spin blockade[5]. For taking the data in **g**, a MW burst with frequency $f_{MW}^{ST}$ and duration $t_{MW}^{ST}$ is applied to the PC

gate at stage F. **e**, The state evolution during the $Q_{ST}$ rotation. The trajectories of $Q_{ST}$ at detunined points marked by the square and star symbols are illustrated in the laboratory-frame Bloch spheres shown on the right. **f**, FFT spectra of the data in **e**. The white dashed curve is an eye guide of the spectral peaks drawn at $\sqrt{J^{\mathrm{ST}^2} + \Delta E_{\mathrm{Z}}^{\mathrm{ST}^2}}/h$. **g**, A MW frequency dependence of resonantly driven rotation, taken at the point shown by the black triangle in **c**. **h**, Rabi oscillation of $Q_{ST}$ measured at the resonance, $f_{\mathrm{MW}}^{\mathrm{ST}} = 345$ MHz.

**Supplementary Note 3: The origin of the two-qubit gate.**

In this section we discuss the conditions for Eq. 1 to be a good approximation[7]. The general Hamiltonian of the three-spin system in the (1,1,1) charge state under a magnetic field is given by

$$\mathcal{H} = \mathcal{H}_Z + \mathcal{H}_J \qquad (1)$$

$$\mathcal{H}_Z = -E_{\mathrm{Z}}\hat{\sigma}_z^{\mathrm{LD}}/2 - (\Delta E_{\mathrm{Z}}^{\mathrm{QQ}} + E_{\mathrm{Z}})\hat{\sigma}_z^{\mathrm{C}}/2 - (\Delta E_{\mathrm{Z}}^{\mathrm{ST}} + \Delta E_{\mathrm{Z}}^{\mathrm{QQ}} + E_{\mathrm{Z}})\hat{\sigma}_z^{\mathrm{R}}/2 \qquad (2)$$

$$\mathcal{H}_J = J^{\mathrm{QQ}}(\hat{\boldsymbol{\sigma}}^{\mathbf{LD}} \cdot \hat{\boldsymbol{\sigma}}^{\mathbf{C}} - \mathbf{1})/4 + J^{\mathrm{ST}}(\hat{\boldsymbol{\sigma}}^{\mathbf{C}} \cdot \hat{\boldsymbol{\sigma}}^{\mathbf{R}} - \mathbf{1})/4 \qquad (3)$$

where $\mathcal{H}_Z$ and $\mathcal{H}_J$ represent the Zeeman energy term and the exchange coupling, respectively. Here $\hat{\boldsymbol{\sigma}}^{\mathbf{LD}}$ ($\hat{\sigma}_z^{\mathrm{LD}}$), $\hat{\boldsymbol{\sigma}}^{\mathbf{C}}$ ($\hat{\sigma}_z^{\mathrm{C}}$) and $\hat{\boldsymbol{\sigma}}^{\mathbf{R}}$ ($\hat{\sigma}_z^{\mathrm{R}}$) are the Pauli operators (and their *z*-component) of the spin in the left, center and right dots, respectively. $E_{\mathrm{Z}}$ is the Zeeman energy of the spin in the left dot. $\Delta E_{\mathrm{Z}}^{\mathrm{QQ}}$ and $\Delta E_{\mathrm{Z}}^{\mathrm{ST}}$ are the Zeeman energy difference between the left and center dots and between the right and center dots, respectively. We assume exchange couplings only between neighboring dots, $J^{\mathrm{QQ}}$ ($J^{\mathrm{ST}}$) between the left (right) and center dots. The Zeeman energy difference between the neighboring dots $\Delta E_{\mathrm{Z}}^{\mathrm{QQ}}$ ($\Delta E_{\mathrm{Z}}^{\mathrm{ST}}$) competes with the exchange coupling $J^{\mathrm{QQ}}$ ($J^{\mathrm{ST}}$). In the case of $\Delta E_{\mathrm{Z}}^{\mathrm{QQ}}, \Delta E_{\mathrm{Z}}^{\mathrm{ST}} \gg J^{\mathrm{QQ}}, J^{\mathrm{ST}}$ which is the case in our experiments, the three-spin eigenstates are well approximated by three isolated spins $|\sigma_z^{\mathrm{LD}}\sigma_z^{\mathrm{C}}\sigma_z^{\mathrm{R}}\rangle$ rather than the exchange dominated states of the doublets and quardruplets[8]. Those three-spin states, mainly determined by $\mathcal{H}_Z$, are perturbed by $\mathcal{H}_J$. At the two-qubit interaction point which is near the resonance of (1,1,1) and (2,0,1) charge state, $J^{\mathrm{QQ}} \gg J^{\mathrm{ST}} \sim 0$ is satisfied and therefore the second term of Supplementary Eq. 3 is neglected. Furthermore, we apply large external magnetic field such that $E_{\mathrm{Z}} \gg \Delta E_{\mathrm{Z}}^{\mathrm{QQ}}, \Delta E_{\mathrm{Z}}^{\mathrm{ST}}$ to energetically separate $|\uparrow\uparrow\uparrow\rangle$ and $|\downarrow\downarrow\downarrow\rangle$ from the two-qubit subspace of $|\uparrow\uparrow\downarrow\rangle, |\uparrow\downarrow\uparrow\rangle, |\downarrow\uparrow\downarrow\rangle$ and $|\downarrow\downarrow\uparrow\rangle$, with the consequence that the leakage from the qubit states to those fully spin-polarized states becomes negligible. The state leakage to the other non-qubit states, $|\downarrow\uparrow\uparrow\rangle$ and $|\uparrow\downarrow\downarrow\rangle$, is also suppressed by adiabatically turning on and off $J^{\mathrm{QQ}}$ with respect to $\Delta E_{\mathrm{Z}}^{\mathrm{QQ}}$[9]. Therefore we can restrict ourselves only to the two-qubit subspace under the condition of $E_{\mathrm{Z}} \gg \Delta E_{\mathrm{Z}}^{\mathrm{QQ}}, \Delta E_{\mathrm{Z}}^{\mathrm{ST}} \gg J^{\mathrm{QQ}} \gg J^{\mathrm{ST}}$. Here the first, second and third terms of Supplementary Eq. 2 are simplified to the first and the second terms of Eq. 1. In addition, the first term of Supplementary Eq. 3 can be approximated by the last term of Eq. 1. In our experiment, the relevant parameter values at the two-qubit interaction configuration are $E_{\mathrm{Z}}/h \sim 17$ GHz, $\Delta E_{\mathrm{Z}}^{\mathrm{QQ}}/h \sim$

$\Delta E_{\mathrm{Z}}^{\mathrm{ST}}/h \sim 0.5$ GHz, $J^{\mathrm{QQ}}/h \sim 0.09$ GHz and $J^{\mathrm{ST}}/h \sim 0$ GHz and therefore the above conditions are satisfied and Eq. 1 is a good approximation.

**Supplementary Note 4: The controllability of $J^{\mathrm{QQ}}$ by the gate voltages.**

In this section we discuss the three-spin state energy diagram in our experimental setup and its gate voltage dependence. Supplementary Fig. 3a shows the calculated energy levels as a function of the gate voltages $V_{\mathrm{PL}}$ and $V_{\mathrm{PR}}$ which are changed to detune the energies between the outer dots while keeping the center dot energy level fixed[1,8]. Here we assume a common inter-dot tunnel coupling $t$ between neighboring dots and Zeeman energies $E_{\mathrm{Z}}/h = 17$ GHz, $\Delta E_{\mathrm{Z}}^{\mathrm{QQ}}/h = \Delta E_{\mathrm{Z}}^{\mathrm{ST}}/h = 0.5$ GHz. The red and blue curves in Supplementary Fig. 3c show the $Q_{\mathrm{LD}}$ state dependent ST precession frequencies $f_{\sigma_z^{\mathrm{LD}}}^{\mathrm{ST}}$ as a function of $V_{\mathrm{PL}}$. Supplementary Fig. 3b shows the $f_{\sigma_z^{\mathrm{LD}}}^{\mathrm{ST}}$ spectra measured at the three interaction points marked by the star, triangle and square symbols in Fig. 1b. Here we obtain the spectra by Bayesian estimation[3,10] (see also Methods) instead of the FFT used in Fig. 2c. By fitting the spectra, we obtain splittings of $f_{|\downarrow\rangle}^{\mathrm{ST}}$ and $f_{|\uparrow\rangle}^{\mathrm{ST}}$, $f_{|\downarrow\rangle}^{\mathrm{ST}} - f_{|\uparrow\rangle}^{\mathrm{ST}} = 6.08 \pm 0.01, 17.18 \pm 0.01$ and $63.13 \pm 0.01$ MHz for each point. These values are plotted in Fig. 2d. Then we fit the obtained gate voltage dependence of $f_{|\downarrow\rangle}^{\mathrm{ST}} - f_{|\uparrow\rangle}^{\mathrm{ST}}$ with the model curve calculated from Supplementary Fig. 3a as shown by the black curve. We find an agreement between the theory model and the data upon choosing $t = 1.2$ GHz and the lever arm of the detuning energy against $V_{\mathrm{PL}}$ of 18 meV/V.

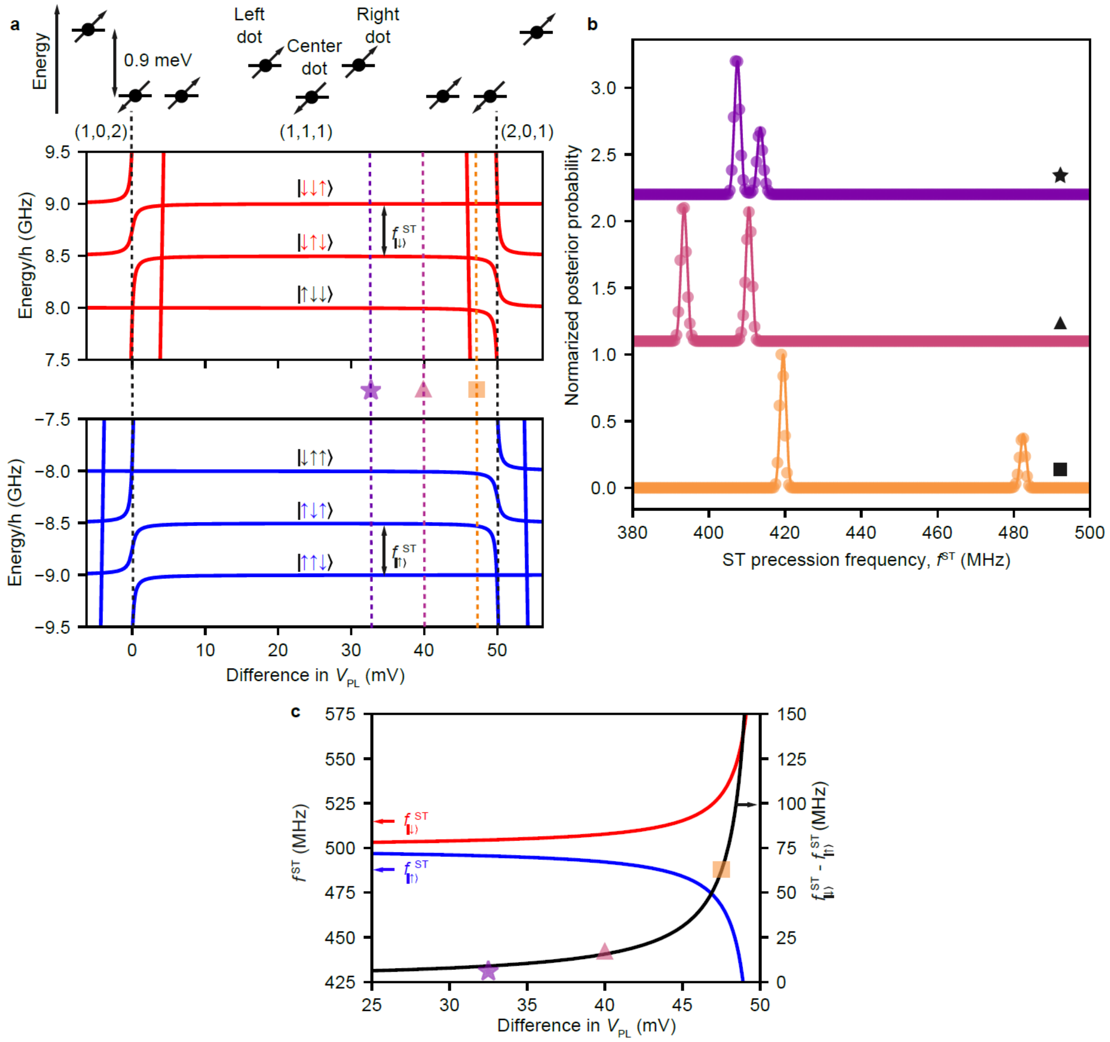


**Supplementary Figure 3 | Three-spin state energy diagram.**

**a**, Three-spin state energy diagram as a function of $V_{\mathrm{PL}}$ with a corresponding energy level of each dot. The voltages are plotted as difference from the degeneracy of (1,1,1) and (1,0,2) charge states, at $(V_{\mathrm{PL}}, V_{\mathrm{PR}}) =$ (-348 mV, -208 mV) (see Fig. 1b). The degeneracy between (1,1,1) and (2,0,1) charge states is located at $(V_{\mathrm{PL}}, V_{\mathrm{PR}}) =$ (-298 mV, -248 mV). We assume that the energy difference between (1,0,2) and (2,0,1) charge states, corresponding to the difference in $(V_{\mathrm{PL}}, V_{\mathrm{PR}})$ being (50 mV, -40 mV), is 0.9 meV. This gives an agreement with the measured data (three symbols) in Fig. 2d. The positions of the interaction points used in Figs. 2c and 2d are shown by dashed lines with the corresponding symbols. **b**, Posterior probability of $f^{\mathrm{ST}}$ calculated by Bayesian estimation from the data used in Fig. 2c (traces offset for clarity). Each trace is normarized by its maximum. The solid curves are the fit with the weighted sum of two Gaussian distributions. **c**, $f^{\mathrm{ST}}_{\sigma_z^{\mathrm{LD}}}$ (the red and blue curves) and $f^{\mathrm{ST}}_{|\downarrow\rangle} - f^{\mathrm{ST}}_{|\uparrow\rangle}$ (the black curve) extracted from **a** as a function of $V_{\mathrm{PL}}$. The three symbols are obtained from **b**.

**Supplementary Note 5: The model of the ST precession under $J^{\mathrm{QQ}}$.**

Here we explain the ST precession model $\mathrm{P}_{\mathrm{S,model}}$ in Eq. 2. We assume that $\mathrm{P}_{\mathrm{S,model}}$ is a Gaussian decaying oscillation function[11] with an oscillation frequency $f^{\mathrm{ST}}_{\sigma_z^{\mathrm{LD}}}$. The decay time $T_2^*$ is the dephasing time of $\mathrm{Q_{ST}}$ determined by fluctuating $\phi^{\mathrm{ST}}$ (due to nuclear field and charge noise) occuring within a single pulse cycle of 700 μs (in the black dashed square in Fig. 2b). The oscillations amplitude $a = 0.218$ and the mean value of the oscillation $b = 0.511$ are determined by three factors, i.e., initialization error of $\mathrm{Q_{ST}}$, tilt of the precession axis determined by $J^{\mathrm{ST}}/\Delta E_{\mathrm{Z}}^{\mathrm{ST}}$ and the readout error of $\mathrm{Q_{ST}}$. The phase of $\mathrm{Q_{ST}}$ is described by two terms, $\mathrm{Q_{LD}}$-controlled phase $\phi_{\sigma_z^{\mathrm{LD}}} = -\pi\sigma_z^{\mathrm{LD}}J^{\mathrm{QQ}}(t_{\mathrm{int}} + t_0)/h$ and the single-qubit phase $\phi^{\mathrm{ST}} = 2\pi\Delta E_{\mathrm{Z}}^{\mathrm{ST}}(t_{\mathrm{int}} + t_{\mathrm{ramp}})/h + \phi_0$ which accumulates independently from $\mathrm{Q_{LD}}$. Here $t_0$ accounts for the effective total time for turning on and off $J^{\mathrm{QQ}}$ with voltage pulses. The estimated value of $t_0 = 1.53$ ns found by MLE is much smaller than the voltage pulse ramp time of $t_{\mathrm{ramp}} = 24$ ns as $J^{\mathrm{QQ}}$ rapidly increases only near the resonance of (1,1,1) and (2,0,1) charge states (see Fig. 2d). Since $\Delta E_{\mathrm{Z}}^{\mathrm{ST}}$ also varies during the voltage ramps due to the inhomogeneity of the MM-induced magnetic field, an additional phase $\phi_0$ is necessary to properly describe $\phi^{\mathrm{ST}}$. We note the fluctuation of $\phi_0$ is independent from that of $\Delta E_{\mathrm{Z}}^{\mathrm{ST}}$ due to the inhomogeneity of the nuclear Overhauser field. In this model, any possible fluctuation of $J^{\mathrm{QQ}}$ due to charge noise is equivalent to an additional fluctuation in $\Delta E_{\mathrm{Z}}^{\mathrm{ST}}$ and $\phi_0$, and therefore does not have to be parametrized separately.

**Supplementary Note 6: Coherence of the two qubits.**

Here we discuss possible limiting factors of $T_2^*$ in Eq. 2 following the analysis presented in Ref. 10. $T_2^*$ is the dephasing time caused by the two fluctuators: the nuclear field and charge noise, within the data acquisition time. To evaluate the characteristic time scale of the fluctuation, we calculate the time correlator of $f^{\mathrm{ST}}_{\sigma_z^{\mathrm{LD}}}$, $C_f(\Delta t) = f^{\mathrm{ST}}_{\sigma_z^{\mathrm{LD}}}(t + \Delta t) - f^{\mathrm{ST}}_{\sigma_z^{\mathrm{LD}}}(t)$ as a function of time delay, $\Delta t$ (see Ref. 10). The histogram of the correlator has a main peak at $C_f = 0$ MHz and two side peaks at $\pm J^{\mathrm{QQ}}/h = \pm 90$ MHz due to flip-flops of the $\mathrm{Q_{LD}}$ state (Supplementary Fig. 4a). All of the peaks show Gaussian distribution and their variance $\sigma_f^2(\Delta t)$, which has similar values between each peak, determines $T_2^*$ as $T_2^*(\Delta t) \propto 1/\sigma_f(\Delta t)$. Supplementary Fig. 4b shows the $\Delta t$ dependence of $\sigma_f^2$ calculated from the main peak of $C_f$. We identify two different regimes, $50$ ms $< \Delta t$ and $\Delta t < 50$ ms. For $50$ ms $< \Delta t$, the variance shows the dependence $\sigma_f^2 \propto \Delta t^{0.8}$ similar to Ref. 10, where the diffusion of nuclear spins is suggested to be the origin. In this regime, the variance and thereby $T_2^*$ are limited by the Overhauser field fluctuation. On the other hand, in $\Delta t < 50$ ms, the variance displays saturation. This suggests another noise source with a larger high-frequency tail, which we believe is due to the charge noise becoming dominant. Therefore we suppose that in our two-qubit gate experiment, the coherence of $\mathrm{Q_{ST}}$ is limited by charge noise. In this regime, the observed

dephasing time dominates the dephasing of the whole two-qubit system, and therefore we conclude that the dephasing time of our CPHASE gate is $211$ ns.

In contrast, for the ST precession measurement data shown in Figs. 2e and 2f, a much longer data acquisition time of 451 ms results in $T_2^* \approx 70$ ns, limited by the nuclear spins.

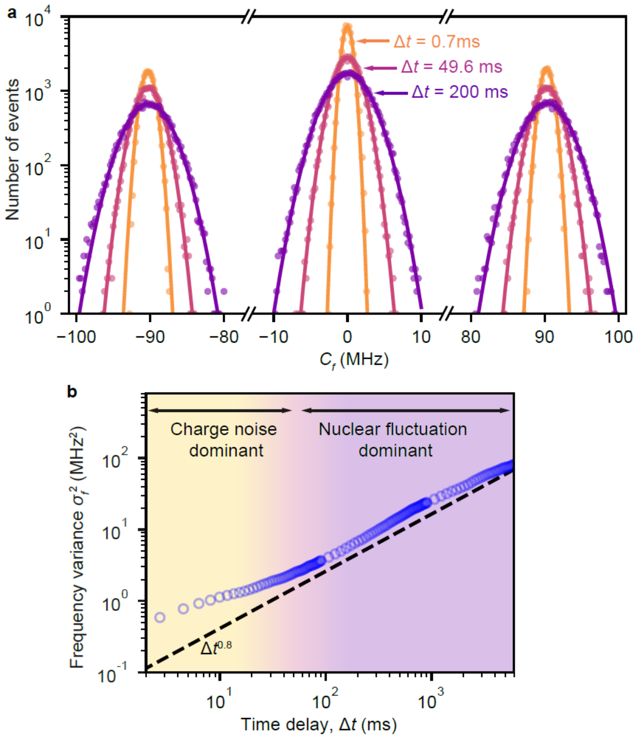


**Supplementary Figure 4 | Variance of the ST precession frequency.**

**a**, Histogram of the time correlator of the estimated ST precession frequency having a main peak at zero frequency and two side peaks at $\pm J^{\mathrm{QQ}}/h$. Each peak fits well to a Gaussian distribution. **b**, $\Delta t$ dependence of the estimated ST precession frequency variance calculated from the main peak of $C_f$ (blue open circles). The dotted line is $\sigma_f^2 \propto \Delta t^{0.8}$ reflecting the nuclear spin diffusion[10].

**Supplementary Note 7: The unconditional phase accumulation during the CPHASE gate.**

Here we describe the condition where the CPHASE gate including the single-qubit phase gates of $Q_{\mathrm{ST}}$ and $Q_{\mathrm{LD}}$ is realized[7,12]. From Eqs. 1 and 2, the two-qubit interaction in the basis states of $|\uparrow\uparrow\downarrow\rangle, |\uparrow\downarrow\uparrow\rangle, |\downarrow\uparrow\downarrow\rangle$ and $|\downarrow\downarrow\uparrow\rangle$ is given by the unitary operation,

$$U(t_{\mathrm{int}}) = Z^{\mathrm{LD}}(-\phi^{\mathrm{LD}})Z^{\mathrm{ST}}(-\phi^{\mathrm{ST}})\begin{pmatrix}1 & 0 & 0 & 0\\ 0 & e^{-i\phi_{|\uparrow\rangle}} & 0 & 0\\ 0 & 0 & e^{i\phi_{|\downarrow\rangle}} & 0\\ 0 & 0 & 0 & 1\end{pmatrix}$$

$$= Z^{\mathrm{LD}}\left(-\phi^{\mathrm{LD}} + \phi_{|\downarrow\rangle}\right)Z^{\mathrm{ST}}\left(-\phi^{\mathrm{ST}} - \phi_{|\uparrow\rangle}\right)\begin{pmatrix}1 & 0 & 0 & 0\\ 0 & 1 & 0 & 0\\ 0 & 0 & 1 & 0\\ 0 & 0 & 0 & e^{-i(\phi_{|\downarrow\rangle}-\phi_{|\uparrow\rangle})}\end{pmatrix} \quad (4)$$

where $Z^{\mathrm{LD}}(\phi)$ and $Z^{\mathrm{ST}}(\phi)$ represent qubit rotation around the $z$-axis by angle $\phi$ for $Q_{\mathrm{LD}}$ and $Q_{\mathrm{ST}}$, respectively. Up to single-qubit phases (neglecting $Z^{\mathrm{LD}}(-\phi^{\mathrm{LD}} + \phi_{|\downarrow\rangle})$ and $Z^{\mathrm{ST}}(-\phi^{\mathrm{ST}} - \phi_{|\uparrow\rangle})$), the CPHASE gate is realized for any integer $n$ such that $\phi_{\mathrm{C}} = \phi_{|\downarrow\rangle} - \phi_{|\uparrow\rangle} = 2\pi J^{\mathrm{QQ}}(t_{\mathrm{int}} + t_0)/h = \pi(2n+1)$. This condition is met in our experiment with $t_{\mathrm{int}} = 4.0 + 11n$ ns where 4.0 ns comes from the initial phase due to a finite value for $t_0$.

More strictly, to construct the CPHASE gate without ignoring the single-qubit phases, each single-qubit phase should satisfy $\phi^{\mathrm{LD}} - \phi_{|\downarrow\rangle} = 2\pi l$ and $\phi^{\mathrm{ST}} + \phi_{|\uparrow\rangle} = 2\pi m$ where $l$ and $m$ are integers. This can be realized by a phase gate of $Q_{\mathrm{ST}}$ and $Q_{\mathrm{LD}}$ or a decoupled CPHASE gate[13,14], although we do not perform them in our experiment.

**Supplementary Note 8: The quality factor of the CPHASE gate.**

Here we discuss the performance of the CPHASE gate. A convenient figure of merit is the quality factor $Q$ representing the number of possible CPHASE gate operations within the dephasing time. It is evaluated to be $Q = 2J^{\mathrm{QQ}}T_2^*/h = 38$ in our experiment. For comparison, Ref. 13 uses a two-qubit gate with $Q \sim 10$ and evaluates the lower bound of the gate fidelity to be 85 % extracted from the Bell state tomography. Our value of $Q$ therefore suggests that our CPHASE gate fidelity could be potentially higher than 85 %. However, the gate fidelity has to be characterized by more elaborate approaches such as process tomography or randomized bechmarking in future experiments. Si-based devices with much better single-qubit gate fidelity will allow these detailed characterizations.

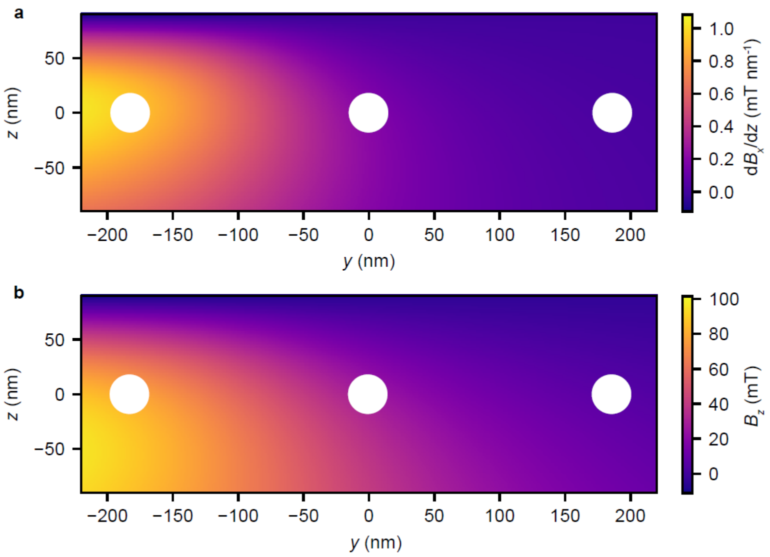


**Supplementary Figure 5 | Local magnetic field simulation.**

**a**, Simulated distribution of the slanting field $\mathrm{d}B_x/\mathrm{d}z$ created by a MM for the design shown in Fig. 1a calculated by the boundary integral method[15]. The white circles indicate the positions of the three dots from the device lithography design. **b**, Simulated local Zeeman field $B_z$.

| $Q_{LD}$ | Interaction time index $k$ / Pulse cycle index $m$ | $k = 1$ ($t_{\text{int}} = 0.83$ ns) | $\cdots$ | $k = 100$ ($t_{\text{int}} = 83$ ns) |
|---|---|---|---|---|
| $\sigma_z^{\text{LD}} = \|\uparrow\rangle$ | $m = 1$ | $r_{m=1}^{k=1}$ | $\cdots$ | $r_{m=1}^{k=100}$ |
| | $\vdots$ | $\vdots$ | $\cdots$ | $\vdots$ |
| | $m = 2000$ | $r_{m=2000}^{k=1}$ | $\cdots$ | $r_{m=2000}^{k=100}$ |
| $\sigma_z^{\text{LD}} = \|\downarrow\rangle$ | $m = 2001$ | $r_{m=2001}^{k=1}$ | $\cdots$ | $r_{m=2001}^{k=100}$ |
| | $\vdots$ | $\vdots$ | $\cdots$ | $\vdots$ |
| | $m = 4000$ | $r_{m=4000}^{k=1}$ | $\cdots$ | $r_{m=4000}^{k=100}$ |

**Supplementary Table 1 | Data structure for the ST precession parameter estimation.**

Collected data set for the ST precession parameter estimation. $m$, $k$ and $r_m^k$ reperesent the pulse cycle index, the interaction time index $t_{\text{int}}(k) = 0.83k$ ns and the readout result of $Q_{ST}$: $r_m^k = 1$ (0) for $Q_{ST}$ = $|\text{S}\rangle$ ($|\text{T}\rangle$). For each interaction time and $Q_{LD}$ state, we have 2000 readout results whereas for each pulse cycle, we have 100 readout results of $Q_{ST}$.

| Parameter | Minimum | Maximum | Numbre of discretized points | Pulse-cycle-dependency |
|---|---|---|---|---|
| $a$ | 0.208 | 0.223 | 16 | No |
| $b$ | 0.501 | 0.516 | 16 | |
| $J^{\text{QQ}}/h$ (MHz) | 89.8 | 91.3 | 16 | |
| $t_0$ (ns) | 1.4 | 1.7 | 16 | |
| $T_2^*$ (ns) | 160 | 310 | 16 | |
| $\sigma_z^{\text{LD}}$ | $-1$ | 1 | 2 | Yes |
| $\phi_0$ | $\pi/16$ | $2\pi$ | 32 | |
| $\Delta E_Z^{\text{ST}}/h$ (MHz) | 465 | 496 | 32 | |

**Supplementary Table 2 | Parameter space for Equation 3.**

The eight dimensional parameter space for evaluating Eq. 3. The search ranges of the five pulse-cycle-independent parameters are determined based on prior, coarse estimation results over wide spans.

**Supplementary References:**

[1]Noiri, A., Yoneda, J., Nakajima, T., Otsuka, T., Delbecq, M. R., Takeda, K., Amaha, S., Allison, G., Ludwig, A., Wieck, A. D., and Tarucha, S. Coherent electron-spin-resonance manipulation of three individual spins in a triple quantum dot. *Appl. Phys. Lett.* **108**, 153101 (2016).

[2]Shafiei, M., Nowack, K. C., Reichl, C., Wegscheider, W., and Vandersypen, L. M. K. Resolving Spin-Orbit- and Hyperfine-Mediated Electric Dipole Spin Resonance in a Quantum Dot. *Phys. Rev. Lett.* **110**, 107601 (2013).

[3]Shulman, M. D., Harvey, S. P., Nichol, J. M., Bartlett, S. D., Doherty, A. C., Umansky, V., and Yacoby, A. Suppressing qubit dephasing using real-time Hamiltonian estimation. *Nat. Comm.* **5**, 5156 (2014).

[4]Nichol, J. M., Orona, L. A., Harvey, S. P., Fallahi, S., Gardner, G. C., Manfra, M. J., and Yacoby, A. High-fidelity entangling gate for double-quantum-dot spin qubits. *npj Quantum Information* **3**, 3 (2017).

[5]Petta, J. R. *et al*. Coherent Manipulation of Coupled Electron Spins in Semiconductor Quantum Dots. *Science* **309,** 2180-2184 (2005).

[6]Higginbotham, A. P., Kuemmeth, F., Hanson, M. P., Gossard, A. C., and Marcus, C. M., Coherent Operations and Screening in Multielectron Spin Qubits. *Phys. Rev. Lett.* **112**, 026801 (2014).

[7]Mehl, S. and DiVincenzo, D. P. Simple operation sequences to couple and interchange quantum information between spin qubits of different kinds. *Phys. Rev. B* **92,** 115448 (2015).

[8]Laird, E. A., Taylor, J. M., DiVincenzo, D. P., Marcus, C. M., Hanson, M. P., and Gossard, A. C. Coherent spin manipulation in an exchange-only qubit. *Phys. Rev. B* **82**, 075403 (2010).

[9]Nakajima, T., Delbecq, M. R., Otsuka, T., Amaha, S., Yoneda, J., Noiri, A., Takeda, K., Allison, G., Ludwig, A., Wieck, A. D., and Tarucha, S. Coherent transfer of electron spin correlations assisted by dephasing noise. *Nature Communications* **9**, 2133 (2018).

[10]Delbecq, R. M., Nakajima, T., Stano, P., Otsuka, T., Amaha, S., Yoneda, J., Takeda, K., Allison, G., Ludwig, A., Wieck, A. D., and Tarucha, S. Quantum Dephasing in a Gated GaAs Triple Quantum Dot due to Nonergodic Noise. *Phys. Rev. Lett.* **116**, 046802 (2016).

[11]Dial, O. E., Shulman, M. D., Harvey, S. P., Bluhm, H., Umansky, V., and Yacoby, A. Charge Noise Spectroscopy Using Coherent Exchange Oscillations in a Singlet-Triplet Qubit. *Phys. Rev. Lett.* **110**, 146804 (2013).

[12]Meunier, T., Calado, V. E., and Vandersypen, L. M. K. Efficient controlled-phase gate for single-spin qubits in quantum dots. *Phys. Rev. B* **83**, 121403(R) (2011).

[13]Watson, T. F., Philips, S. G. J., Kawakami, E., Ward, D. R., Scarlino, P., Veldhorst, M., Savage, D. E., Lagally, M. G., Friesen, M., Coppersmith, S. N., Eriksson, M. A., and Vandersypen, L. M. K. A programmable two-qubit quantum processor in silicon. *Nature* **555**, 633–637 (2018).

[14]Shulman, M. D., Dial, O. E., Harvey, S. P., Bluhm, H., Umansky, V., Yacoby, A. Demonstration of Entanglement of Electrostatically Coupled Singlet-Triplet Qubits. *Science* **336**, 202-205 (2012).
[15]We used MATHEMATICA RADIA package available at http://www.esrf.fr/ for MM field calculations.
