# Supplementary material for: A fast quantum interface between different spin qubit encodings
Source: Nat Commun. 2018 Nov 29;9:5066. doi: 10.1038/s41467-018-07522-1 (PMC6265340; doi:10.1038/s41467-018-07522-1)
Supplement: Supplementary file 2 — Peer Review file [file 41467_2018_7522_MOESM2_ESM.pdf]

Editorial Note: This manuscript has been previously reviewed at another journal that is not operating a transparent peer review scheme. This document only contains reviewer comments and rebuttal letters for versions considered at Nature Communications.

We thank the reviewer for corroborating the significance of our result. As there is no remaining issue raised by the reviewer, we believe that our manuscript is now appropriate for publication in *Nature Communications*.

Response to Reviewer #4

--

> *While of course it remains to be seen whether the hybrid scheme that this paper presents will be of value, I think bringing together these two main spin qubit variants is a significant result. Following on from my previous review, while I wasn't quite convinced this paper met the threshold for publication in [redacted], I would recommend publication in Nature Communications.*

We thank the reviewer for his/her careful consideration on the manuscript, as well as for helpful suggestions to clarify the manuscript.
